# Aortic Stenosis Phenotypes and Precision Transcatheter Aortic Valve Implantation

**DOI:** 10.3390/jcdd10070265

**Published:** 2023-06-21

**Authors:** Muzamil Khawaja, Hafeez Ul Hassan Virk, Dhrubajyoti Bandyopadhyay, Mario Rodriguez, Johao Escobar, Mahboob Alam, Hani Jneid, Chayakrit Krittanawong

**Affiliations:** 1 Division of Cardiology, Emory University School of Medicine, Atlanta, GA 30322, USA; 2 Harrington Heart & Vascular Institute, Case Western Reserve University, University Hospitals Cleveland Medical Center, Cleveland, OH 44106, USA; 3 Department of Cardiology, Westchester Medical Centre, New York Medical College, Valhalla, NY 10595, USA; 4 Division of Cardiology, Barnes-Jewish Hospital at Washington University in St. Louis School of Medicine, Saint Louis, MO 63110, USA; 5 Division of Cardiology, Harlem Cardiology, New York, NY 10035, USA; 6 Division of Cardiology, The Texas Heart Institute, Baylor College of Medicine, Houston, TX 77030, USA; 7 Division of Cardiology, University of Texas Medical Branch, Houston, TX 77030, USA; 8 Cardiology Division, NYU Langone Health and NYU School of Medicine, New York, NY 10016, USA

**Keywords:** aortic valve stenosis, TAVR, TAVI

## Abstract

Patients with a clinical indication for aortic valve replacement can either undergo surgical aortic valve replacement (SAVR) or Transcatheter Aortic Valve Implantation (TAVI). There are many different factors that go into determining which type of replacement to undergo, including age, life expectancy, comorbidities, frailty, and patient preference. While both options offer significant benefits to patients in terms of clinical outcomes and quality of life, there is growing interest in expanding the indications for TAVI due to its minimally invasive approach. However, it is worth noting that there are several discrepancies in TAVI outcomes in regards to various endpoints, including death, stroke, and major cardiovascular events. It is unclear why these discrepancies exist, but potential explanations include the diversity of etiologies for aortic stenosis, complex patient comorbidities, and ongoing advancements in both medical therapies and devices. Of these possibilities, we propose that phenotypic variation of aortic stenosis has the most significant impact on post-TAVI clinical outcomes. Such variability in phenotypes is often due to a complex interplay between underlying comorbidities and environmental and inherent patient risk factors. However, there is growing evidence to suggest that patient genetics may also play a role in aortic stenosis pathology. As such, we propose that the selection and management of TAVI patients should emphasize a precision medicine approach.

## 1. Introduction

Aortic valve stenosis (AS) is the most common valvular heart disease in developed countries, and its impact on public health and healthcare resources is expected to increase due to aging Western populations [1,2]. Its prevalence steadily increases with age, ranging from 0.2 percent at ages 50 to 59 years, to 1.3 percent at ages 60 to 69, 3.9 percent at ages 70 to 79 years, and 9.8 percent at ages 80 to 89 years [3]. Early recognition and management of AS are crucial because mortality in patients with AS dramatically increases after the development of cardiac symptoms [4]. Unfortunately, valve replacement is the lone effective therapeutic option for patients with AS because no medical therapy has been proven to be efficacious at slowing disease progression or improving clinical outcomes [5]. Prior to the advent of minimally invasive transcatheter aortic valve implantation (TAVI), surgical aortic valve replacement (SAVR) was always considered the gold standard for AS treatment. At the same time, little is known about the ideal timing for valve replacement and which patients may represent poor candidates for replacement, especially when medical comorbidities are taken into consideration. These continued issues are likely secondary to the fact that AS patients are often viewed through a more homogenous lens than what may be warranted. Rather than focusing on hemodynamic indices of valve obstruction, there is growing evidence now to suggest that AS patients are far more heterogeneous with respect to multiple factors including age, comorbidities, frailty, physical function, cardiac remodeling, and function. This paper seeks to explore the pathophysiologic complexity and heterogeneity that exists within AS patients and to evaluate the data for TAVI in patients with such AS phenotypic variation. 

AS, in basic terms, is defined as a narrowing of the aortic valve opening. There are multiple etiologies for AS that contribute in part to phenotypic variation, including congenital (bicuspid/unicuspid), calcific, and rheumatic disease. At the same time, there is also variability in terms of symptomatology in AS patients. The common symptoms of AS include shortness of breath, syncope, and angina, but presentations are highly variable due to the fact that severe AS may present more subtly and initially experience a decrease in exertional tolerance without recognizing classic symptoms [6]. Others may have a more acute presentation. Such symptom heterogeneity often correlates with the hemodynamic severity of AS, but there is also the possibility that concurrent medical conditions can contribute. 

According to the American College of Cardiology/American Heart Association (ACC/AHA) 2020 Guidelines for Management of Valvular Disease [7], transthoracic echocardiography (TTE) is indicated to assess the underlying cause of AS, to evaluate hemodynamic severity, to quantify left ventricular size and systolic function, and to determine overall prognosis and optimal timing of valve intervention. The 2020 ACC/AHA Valvular Heart Disease Guidelines define the sequential stages of AS according to valve anatomy, valve hemodynamics, hemodynamic consequences of AS, and symptoms. Stage A is defined as being at risk of AS with an aortic max velocity (V_max_) < 2 m/s with normal leaflet motion in the absence of symptoms of hemodynamic consequences. This includes patients with bicuspid aortic valve (or other congenital aortic valve anomaly), aortic sclerosis or other risk factors for aortic valve disease. Stage B is classified as progressive AS with mildly to moderately calcified valve leaflets, mildly to moderately reduced valve leaflet mobility, and mild (aortic V_max_ 2.0 to 2.9 m/s or a mean transvalvular pressure gradient < 20 mmHg) or moderate (aortic V_max_ 3.0 to 3.9 m/s or a mean transvalvular pressure gradient of 20 to 39 mmHg) AS. Stage C is defined as severe (severe leaflet calcification/thickening with reduced leaflet motion and an aortic velocity ≥ 4 m/s; no specific valve area required) AS without symptoms. Stage C is further classified into C1 (left ventricle ejection fraction [EF] is normal) and C2 (EF < 50%). Lastly, stage D refers to severe, symptomatic AS. This is further subcategorized into D1 (severe high-gradient AS with aortic V_max_ ≥ 4 m/s or mean transvalvular pressure gradient is ≥40 mmHg), D2 (symptomatic severe low flow, low gradient AS with reduced EF and an aortic valve area ≤ 1.0 cm^2^ with resting aortic V_max_ < 4 m/s or mean pressure gradient < 40 mmHg) and D3 (symptomatic severe low-gradient AS with normal EF; aortic valve area ≤ 1.0 cm^2^ with aortic V_max_ < 4 m/s or mean transvalvular pressure gradient < 40 mmHg). 

This valve and symptom-oriented approach to capturing the diverse phenotypes of AS is insufficient, however, given that the clinical course of AS is variable with complex interactions between a patient’s characteristics and associated cardiac and noncardiac diseases. Moreover, there is growing evidence that extravalvular cardiac damage, such as myocardial fibrosis, can be substantially diverse among AS and associated with adverse outcomes [8]. Such factors imply that staging alone may not comprehensively capture the heterogeneity within AS, and sophisticated phenotyping with multiple factors may have additional value. One study sought to investigate the heterogeneity of AS subgroups with different prognostic significances via the use of cluster analysis [9]. Three groups with several characteristics were identified. Group 1, which comprised 60 individuals, primarily exhibited signs of cardiac dysfunction. On the other hand, group 2 included 86 elderly individuals with various underlying health conditions, particularly end-stage renal disease. Group 3, consisting of 252 individuals, did not show any signs of cardiac dysfunction or comorbidities. Despite similar levels of AS severity, there were notable differences in negative outcomes among the groups over a median 2.4-year monitoring period. The mortality rate was significantly higher for group 1 at 13.3%, compared to 19.8% for group 2 and 6.0% for group 3 (*p* < 0.001). Specifically, when compared to group 3, group 1 exhibited a higher risk of cardiac-related deaths (adjusted hazard ratio: 7.37 [95% CI, 2.00–27.13]; *p* = 0.003), whereas cluster 2 had a greater risk of non-cardiac mortality (adjusted hazard ratio: 3.35 [95% CI, 1.26–8.90]; *p* = 0.015). This ultimately introduces a fresh perspective on categorizing AS patients, considering comorbidities and cardiac dysfunction beyond the valve. Ultimately, such significant phenotypic heterogeneity in AS warrants a proper understanding of such variability and how it may or may not affect clinical outcomes in the setting of treatment with TAVI.

The development of TAVI has been proven to be highly useful in AS patients, providing both improvement in symptoms and statistically significant mortality benefits. Both the current ACC/AHA and European Society of Cardiology/European Association for Cardiothoracic Society (ESC/EACTS) guidelines attempt to identify optimal patients for intervention. Both guidelines recognize that any decision to treat AS with TAVI must consider patient factors (i.e., age, life expectancy, age, and comorbidities), procedural factors, and device factors [7,10]. Therefore, patients with severe AS that are potential candidates for valve replacement should undergo a thorough pre-procedural assessment. This includes evaluation by a multidisciplinary valve team that assesses the patient’s anticipated life expectancy with SAVR or TAVI and the potential benefit for the patient’s quality of life. TAVI is generally preferred in older patients of any surgical risk category with a life expectancy of <10 years, and in older patients at high or prohibitive risk for mortality from SAVR with a life expectancy of ≥1 year.

**Figure 1:**
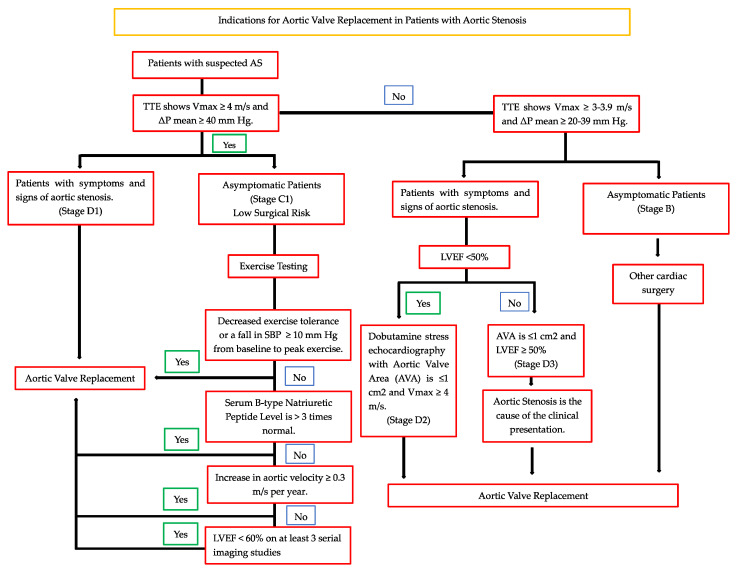
Central illustration.

Currently, the 2020 ACC/AHA [7] indications for aortic valve replacement (either surgical or transcatheter) are as follows (Figure 1):

1. Severe high gradient AS with symptoms (class I recommendation, level B evidence)
2. Asymptomatic patients with severe AS and LVEF < 50 (class I recommendation, level B evidence)
3. Severe AS when undergoing other cardiac surgery (class I recommendation, level B evidence)
4. Asymptomatic severe AS and low surgical risk (class IIa recommendation, level B evidence)
5. Symptomatic with low-flow/low-gradient severe AS (class IIa recommendation, level B evidence)
6. Moderate AS and undergoing other cardiac surgery (class IIa recommendation, level C evidence)

Currently, TAVI is approved for low to prohibitive surgical risk patients with severe AS or valve-in-valve procedures for failed prior bioprosthetic valves [11]. Meanwhile, the 2021 ESC/EACTS Guidelines give a Class IB indication for AS intervention in symptomatic patients with severe gradients or patients with severe low-flow low-gradient AS with reduced EF and evidence of flow reserve [10]. As for asymptomatic AS patients, the ESC/EACTS Guidelines give a Class IB indication for intervention in severe AS and LV dysfunction or severe AS with provocable symptoms on exercise testing [10]. When deciding between SAVR and TAVI, the ESC/EACTS Guidelines give a class IA indication for TAVI in patients older than 75 years of age or at high surgical risk and a class IB indication for SAVR in younger patients with low surgical risk. However, for any patients that do not fit either of these criteria, the decision for SAVR vs. TAVI should be based on individual anatomic, clinical, and procedural characteristics (Class IB) [10]. 

It is also worth mentioning the absolute contraindications for TAVI. These include an estimated life expectancy of <12 months, unlikely improvement in quality of life, additional separate valvular disease with a major contribution to the patient’s symptoms, inadequate aortic annulus size, active endocarditis, and elevated risk of coronary ostium obstruction due to anatomic features [11]. As such, for any patient that may be a candidate for TAVI, the multidisciplinary heart valve team must take into consideration a patient’s life expectancy, frailty, comorbidities, specific anatomy, values, and preferences. Such characteristics ultimately factor into each patient’s unique aortic stenosis phenotype and the probability of success with TAVI. The varieties of aortic stenosis phenotypes and the evidence for or against TAVI use in such circumstances are discussed below (Table 1).

## 2. Molecular Mechanisms and TAVI

As previously mentioned, AS is a progressive disease characterized by abnormal fibrosis and/or calcification of aortic valve leaflets, resulting in disrupted blood flow. Further investigations into the pathogenesis of AS have found links to proatherogenic risk factors, flow-induced mechanical forces, and disease-prone environmental influences [18]. As such, recent breakthroughs in the field of epigenetics offer a new perspective on gene regulation and its role in AS. Four major epigenetic mechanisms: DNA methylation, posttranslational histone modification, ATP-dependent chromatin remodeling, and non-coding regulatory RNAs, contribute to the initiation and progression of AS [18]. Recent data indicate that these mechanisms could contribute to endothelial dysfunction, disease-prone activation of monocyte-macrophage and circulatory osteoprogenitor cells, and osteogenic transdifferentiation of aortic valve interstitial cells, consequently leading to valvular inflammation, fibrosis, and calcification. These mechanisms also contribute to pressure overload-induced maladaptive myocardial remodeling and left ventricular hypertrophy. As such, AS can result in cardiac hypertrophy and fibrosis. Interestingly, randomized clinical trials [19] have suggested that patients receiving TAVI find that TAVI reverses left ventricular hypertrophy and fibrosis in high-risk surgical patients. The exact mechanism behind such a protective effect is thought to be multifold. One of the primary pathways of AS progression to fibrosis involves the activation of tissue-specific fibroblasts to myofibroblasts, which are known to cause abnormal tissue stiffening and subsequent fibrosis upon repeated tissue injury. Repetitive valvular injury activates valvular interstitial cells to become myofibroblasts responsible for tissue homeostasis [20]. However, these myofibroblasts remain persistently active during AS progression and are then accompanied by a pathological stiffening of the valve leaflets. In addition, the added hemodynamic and mechanical stress from progressive AS contributes to myofibroblast activation [21]. Conversely, there is a significant reduction in these hemodynamic and mechanical stresses on the valve following TAVI that can improve left ventricle function and reduce myofibroblast activation [19]. Aguado et al. [22] further investigated the antifibrotic mechanism behind TAVI and found that a systemic inflammatory response to the aortic valve implant influences fibrotic valve tissue remodeling via the regulation of myofibroblasts. Their study found alterations in the post-TAVI serum composition with inflammatory macrophage factors implicated in myofibroblast activation and deactivation. This post-TAVI serum reduced the myofibroblast activation of valvular interstitial cells relative to the pre-TAVI serum from the same patient. The analysis also revealed changes from pre-TAVI to post-TAVI in the myofibroblast phenotype. It also identified the p38 MAPK signaling as being involved in pre-TAVI–mediated myofibroblast activation. Interestingly, post-TAVI serum was found to convert valvular and cardiac myofibroblasts, initially exposed to pre-TAVI serum, to a quiescent fibroblast phenotype. Therefore, this study suggests that alterations in serum composition after TAVI may lead to an antifibrotic fibroblast phenotype, which may correlate with earlier interventions being more beneficial for patients with advanced AS to prevent further disease progression.

## 3. CHIP and TAVI

A more recently discovered potent and independent cardiovascular risk factor possibly associated with AS progression and TAVI outcomes is clonal hematopoiesis of indeterminate potential (CHIP). CHIP refers to the presence of a clonally expanded hematopoietic stem cell line caused by a leukemogenic mutation in individuals without evidence of hematologic malignancy, dysplasia, or cytopenia [23]. CHIP arises from somatic mutations in hematopoietic stem cells that yield mutant leukocytes in the blood. Because of chronic inflammation predisposing to atherogenesis, individuals diagnosed with CHIP (via DNA sequencing) have a higher risk of coronary heart disease, ischemic stroke, and poorer heart failure outcomes independent of traditional cardiovascular risk factors. CHIP can also contribute to AS because of the correlation between chronic inflammation and calcified degenerative AS. In fact, one study examined this underlying link between inflammation, somatic CHIP mutations (i.e., the DNMT3A and TET2 genes) and TAVI outcomes for AS patients [24]. The incidence of DNMT3A- and TET2-CHIP-driver mutations in a cohort of 279 total patients undergoing TAVI for severe calcified AS was assessed to determine whether there was any association between CHIP’s inflammatory blood cell phenotype and clinical outcomes. Even after successful replacement of the stenotic AV by TAVI, the study found that patients carrying a DNMT3A- or TET2-CHIP-driver mutation experienced a significantly worse clinical outcome for death during the first 8 months after the procedure. This association was independent of gender, age, and NT-proBNP serum levels for patients. As for the mechanism behind this increased mortality, myeloid and T-cell line analysis revealed patients harboring a DNMT3A-CHIP-driver mutation in the setting of an increased T helper 17/regular T cell ratio indicating a pro-inflammatory T-cell polarization, whereas patients harboring a TET2-CHIP-driver mutation exhibited increased levels of circulating non-classical monocytes, which are known to secrete high levels of pro-inflammatory cytokines. Although this is the only study to date that has assessed CHIP and TAVI outcomes, it reveals the need to carefully consider every patient’s unique underlying risk factors and how they may contribute to post-TAVI outcomes. 

## 4. Inflammation and TAVI

The role of inflammation in AS and post-TAVI clinical outcomes is an area of growing interest. Inflammation has been found to be tied to TAVIs and AS in a variety of manners. One such inflammatory association is that of post-procedural inflammation after TAVI. Systemic inflammation after TAVI can be associated with increased morbidity and mortality in such a patient population. In fact, some studies estimate that systemic inflammatory response syndrome (SIRS) occurs in as high as approximately 50% of patients after the TAVI procedure [25,26]. However, the exact mechanism behind this inflammation is still unclear. Several potential factors are hypothesized as contributing to this inflammatory response. These include the type of TAVI delivery system, the duration of the procedure, and even the use of iodinated contrast [27]. Nevertheless, significant effort over recent years has gone into improving existing prognostic models for risk stratification of patients undergoing TAVI.

Recent research now suggests that certain inflammatory plasma biomarkers could predict clinical outcomes for TAVI. For instance, elevated levels of high-sensitivity C-reactive protein (hs-CRP) or white blood cells (WBC), as part of SIRS, have been shown to predict outcome and mortality, particularly in the presence of diabetes [28]. Even the neutrophil-to-lymphocyte ratio has been found to be an accurate prognostic clinical marker of systemic inflammatory response in cardiovascular disease patients [29,30,31,32]. One study even investigated the periprocedural course of a common selection of pro- and anti-inflammatory markers in patients with AS, using all currently available aortic valve replacement techniques at a single institution. This study found that, in comparison to surgical aortic valve replacement, transfemoral TAVI was the procedure with the most attenuated inflammatory response [33]. Factors such as the pre-treatment patient condition and the extent of myocardial injury also significantly affected inflammatory biomarker patterns. A separate study found a stronger pro-inflammatory response with IL-6 following procedures with higher surgical invasiveness [34]. Meanwhile, Erdoes et al. described an increase in high-sensitivity CRP and leukocyte counts after SAVR and transapical-TAVI with no fulminant changes observed in Transfemoral-TAVI [33]. Growth differentiation factor-15 is another identified inflammatory marker tied to post-TAVI clinical outcomes, such as all-cause mortality, global longitudinal strain, reduced kidney function, and elevated NT-proBNP levels [35].

One study further evaluated the inflammatory profile differences within TAVI techniques by investigating inflammatory responses after the placement of self-expanding valves versus balloon-expandable valves. Interestingly, self-expanding valves were associated with a more pronounced inflammatory response as manifested by higher white blood cell and neutrophil counts [27]. However, this same study’s variable analysis noted that the duration of the procedure, septum thickness at baseline, and the amount of contrast used were also related to a heightened post-procedural inflammatory response. Clinically, the inflammatory responses with increased WBC post-TAVI were associated with higher rates of major bleeding, arrhythmia, and mortality at 30 days.

Another intriguing matter of interest is regarding the question of whether aortic valve stenosis is accelerated by inflammation or whether it is itself a cause of inflammation. Baratchi et al. [36] explored this topic and found that high shear stress on cells, in the setting of aortic valve stenosis, activates multiple proinflammatory monocyte functions. They assessed inflammatory profiles in patients post TAVI and demonstrated that shear stress-dependent inflammation was downregulated in patients receiving TAVI. Overall, the data regarding inflammatory changes associated with AS and TAVI is often conflicting, and further validation studies are warranted to better understand the pathophysiology.

## 5. TAVI in ESRD and CKD

Another subset of patients with AS that has garnered attention for TAVI is of end-stage renal disease (ESRD) and chronic kidney disease (CKD) patients. This population is of particular interest because studies show that CKD and ESRD patients on hemodialysis (HD) have a higher risk of developing AS at a faster rate and at earlier ages than non-dialysis patients [37]. This occurs because of pathogenic calcium metabolic pathways in CKD and ESRD patients that accelerate aortic valve calcification [38]. Previous studies have also reported an increased risk of mortality for patients with CKD and AS [13]. Moreover, these same patients have an extremely high mortality risk for SAVR, which leaves either TAVI or valvuloplasty as the only other procedural treatment modalities available for severe AS. The data on TAVI in these patients are limited due to CKD and ESRD patients being excluded from the original high-risk patient groups used for regulatory approval studies of TAVI devices. The literature is now growing in this area of concern, but it is notably inconsistent in clinical outcomes. For instance, Bohbot et al. evaluated the effect of TAVI versus conservative management on long-term mortality by stage of CKD and found TAVI dramatically reduced all-cause and cardiovascular mortality at 5 years across all stages of CKD [13]. 

## 6. TAVI in Bicuspid Aortic Valves

AS can also be associated with congenital syndromes. For instance, Turner syndrome is linked to several cardiovascular malformations, such as bicuspid aortic valve, aortic dilatation, or dissection, and coarctation of the aorta. Such aortic stenosis with bicuspid morphology offers a unique therapeutic challenge to the interventional cardiologist and portends an additional risk compared to non-Turner patients due to coexisting aortopathy and coarctation. In such instances, SAVR is the first line of treatment. However, high-risk surgical candidates may benefit from TAVI. There are several other rare genetic syndromes associated with bicuspid aortic valve [39], such as Loeys-Dietz syndrome, Down syndrome, velocardiofacial syndrome, Alagille syndrome, and Kabuki syndrome (Table 1). While these syndromes are associated with bicuspid aortic valves, they have significantly variable degrees of AS, if it develops. It is also notable that most people with bicuspid aortic valves do not have syndromic features, but could have other congenital heart and vascular abnormalities with variable disease severity. There are several novel genes isolated as contributing to bicuspid aortic valve independently of rare genetic syndromes (Table 2). While the spectrum of bicuspid aortic valves is vast, published data regarding TAVI in bicuspid aortic stenosis have been insufficient, mainly relying on a few case series. However, now, more comparative data are becoming available. In a recent study, TAVI was found to be an effective and safe option in bicuspid aortic stenosis [40]. Several case reports have documented success with the use of TAVI in the setting of bicuspid aortic stenosis. Significantly, a comprehensive retrospective analysis was conducted using data from the Society of Thoracic Surgeons/American College of Cardiology Transcatheter Valve Therapy Registry, covering the period from November 2011 to November 2018. The purpose was to investigate the success of the devices, procedural outcomes, post-TAVI valve performance, and clinical outcomes during hospitalization (including mortality, stroke, and major bleeding) based on the type of valve morphology (bicuspid or tricuspid) [12]. When the latest-generation devices were utilized for treating patients with bicuspid aortic valves, the rate of device success improved (93.5% versus 96.3%; *p* = 0.001), and the occurrence of moderate to severe aortic insufficiency decreased (14.0% versus 2.7%; *p* < 0.001) compared to using older-generation devices. However, with the current-generation devices, the rate of device success was slightly lower in the bicuspid aortic valve group compared to the tricuspid group (96.3% in bicuspid versus 97.4% in tricuspid; *p* = 0.07). Additionally, patients with bicuspid aortic valves had a slightly higher incidence of residual moderate or severe aortic insufficiency (2.7% versus 2.1%; *p* < 0.001). Furthermore, in the Medicare-linked cohort, patients with bicuspid aortic valves exhibited a lower adjusted risk of mortality at the one-year mark (hazard ratio: 0.88 [95% CI, 0.78–0.99]), whereas no significant difference was observed in the adjusted risk of stroke within the first year (hazard ratio: 1.14 [95% CI, 0.94–1.39]).

## 7. TAVI in Cirrhosis

Due to the increasing rates of alcohol consumption, hepatitis C, and fatty liver disease, liver cirrhosis has become more prevalent worldwide. Many cardiovascular complications can potentially be associated with liver cirrhosis, which might explain why it is one known risk factor for morbidity and mortality while undergoing cardiac procedures. One cardiovascular complication that is commonly seen among cirrhotic patients is aortic stenosis. Most current data address how to correct this issue in retrospective cohort studies, demonstrating that TAVI has better outcomes for hospitalization, mortality, and postoperative complications than other approaches. In one study, the length of post-surgery stay at the hospital was the most minor, 6.12 (±5.59) days in TAVI patients as compared to 9.06 (±5.02) and 7.08 (±3.65) days in SAVR and mini-SVR patients, respectively [41]. Another study showed that in-hospital mortality after TAVI in patients with a history of cirrhosis compared with those without cirrhosis was not different (1.6% vs. 1.5%; *p* = 0.95) [42]. There was no difference in the odds of hospital mortality in patients with a history of cirrhosis compared with no cirrhosis (OR 0.97; 95% CI 0.24–3.87). After a multivariate regression model, the total hospital charges did not show a difference among patients with cirrhosis and no cirrhosis (*p* = 0.50) [42]. Regarding complications, data have revealed that even though some cirrhotic patients can have the highest average age and a higher incidence of pre-existing comorbidities, postoperative complications such as arrhythmia, hyponatremia, and coagulopathy developed to a lesser extent in TAVI patients [41].

Some results in cirrhotic patients undergoing TAVI might not be associated with the typical Society of Thoracic Surgeons score, but instead with the cirrhosis stage. In one study with 105 cirrhotic patients, the Society of Thoracic Surgeons score had a median value of 3.8% (with a range of 1.5% to 6.9%), while the Model for End-Stage Liver Disease (MELD) score had a median value of 11.6 (ranging from 9.4 to 14.0). The MELD score (adjusted hazard ratio: 1.13; 95% confidence interval: 1.05–1.21; *p* = 0.002) emerged as an independent predictor of long-term survival. In the subgroup of patients with a MELD score below 12, those who underwent TAVI had a lower survival rate compared to those who had SAVR (median survival of 2.8 years versus 4.4 years; *p* = 0.047). However, for patients with a MELD score of 12 or higher, survival rates were similar after TAVI, SAVR, and medical therapy (1.3 years versus 2.1 years versus 1.6 years, respectively; *p* = 0.53) [15].

## 8. TAVI in Pregnancy

Pregnancy and AS are thought to coexist in less than 0.01% [43]. The most common etiology for AS in pregnancy is congenital bicuspid aortic valve followed by other congenital abnormalities or rheumatic heart disease [44,45]. In most cases, the diagnosis of aortic stenosis can inadvertently pass in women because they may not become symptomatic until pregnancy due to hemodynamic changes that occur during this period. However, even though the mortality in pregnant women with AS is very low with the advent of new therapies, this population has an increased risk of heart failure and higher hospitalization rates, particularly with severe AS [46,47]. These risks should be ideally reduced by performing aortic valve replacement before patients become pregnant [46].

Severe AS in pregnancy can cause various maternal and fetal disturbances because of the inability of the stenotic valve to support an adequate heart rate and stroke volume demand [14]. Most of the data on successful TAVI in pregnancy comes from case reports. It is important to note that in pregnant women with severe AS, New York Heart Association (NYHA) class III or IV HF symptoms or hemodynamic deterioration are valid indications for valve intervention [7]. Undoubtedly, an alternative for aortic valve replacement is TAVI, since it is a widely approved procedure for high-risk patients [48]. 

Timing of the procedure is a significant factor that should be considered before performing TAVI in pregnant women. The second trimester is the safest time to conduct TAVI for the mother and the fetus [49,50,51]. The benefit of having TAVI in the second trimester is the perfect equilibrium between the lower risks of spontaneous abortion and premature labor, the increase in cardiac output at 24–28 weeks gestation, and the lower risk of fetal complications related to radiation exposure during this time [14]. 

## 9. TAVI in Nonagenarians

The prevalence of AS increases with age, and the nonagenarian group is gradually growing worldwide due to healthcare improvements. AS has several repercussions on the patient’s survival rate and quality of life [52]. Therefore, despite many discrepancies about how to treat nonagenarians with AS, the topic is becoming more debated due to the benefits and challenges depicted in performing this procedure. TAVI is a highly standardized therapy that can be safely performed with high success, even in very old patients. Although mortality is significantly higher in these patients, most probably due to the intrinsic higher risk profile of the very old patients, the results are still acceptable [53]. Moreover, an association between previous severe renal dysfunction and frailty may significantly predict mid-term mortality after TAVI in nonagenarians [54]. Deharo et al. described that among nonagenarians with AS, patients treated with TAVI had a lower risk of cardiovascular events than matched medically treated patients [16]. 

A literature review that identified 16 observational studies found that nonagenarians exhibited significantly higher rates of short-term mortality (hazard ratio [HR], 95% confidence interval [CI]: 1.48, 1.38–1.59; *p* < 0.001) and long-term mortality (HR, 95% CI: 1.34, 1.24–1.44; *p* < 0.001) compared to their younger counterparts following TAVI [54]. Importantly, complications were observed among nonagenarians who underwent TAVI. There were notable disparities in major and/or life-threatening bleeding (risk ratio [RR], 95% CI: 1.21, 1.05–1.39; *p* = 0.008), stroke (HR, 95% CI: 1.24, 1.11–1.40; *p* < 0.001), and major vascular complications (RR, 95% CI: 2.15, 1.35–3.42; *p* = 0.001) between nonagenarians and younger patients post TAVI. However, the rates of minor vascular complications, myocardial infarction, and permanent pacemaker implantation were similar between the two groups. Despite this slight difference in mortality, TAVI in selected nonagenarians is favorable.

## 10. TAVI in LVAD Patients (Left Ventricle Assist Device)

After a left ventricular assist device (LVAD) implantation, one typical result is the development of aortic regurgitation (AR) [55]. TAVI has become the elective procedure for patients with severe aortic stenosis, with an off-label indication for severe AR [56]. The mechanism behind the development of AR after LVAD has several factors. However, it could be explained by the reduced or absent aortic valve opening, which stimulates the fusion of the valve commissures and alters the valve geometry [57]. 

Data regarding TAVI for AR in patients with LVAD are restricted. One study showed that TAVI was associated with a higher improvement rate of NYHA classification in nine patients. At six months, 89% of patients were alive after hospital discharge [58]. In another study involving seven patients with LVAD who underwent TAVI, five (71%) patients survived over a median follow-up of 9 (IQR 6–23) months. Paravalvular complications after device deployment only caused two deaths [56].

Compared with SAVR, Zaidi et al. demonstrated no statistical significance in mortality and readmission with TAVI. Nevertheless, complications, length of stay, and costs were higher in the SAVR group than in the TAVI group [59]. In another study, there was a significantly higher incidence of the primary composite outcome (in-hospital mortality, stroke, transient ischemic attack, MI, pacemaker implantation, need for open aortic valve surgery, vascular complications, and cardiac tamponade) in patients undergoing SAVR (30%) compared with TAVI (14.3%; *p* = 0.001) [17].

## 11. TAVI in Non-Revascularized Obstructive Coronary Artery Disease

Coronary artery disease (CAD) and AS share analogous risk factors and pathophysiological mechanisms, and they commonly coexist [60,61,62]. The risk for early and late mortality is notably higher in non-revascularized CAD after aortic valve replacement [63,64]. When coronary revascularization is planned, a surgical approach is still the standard of care by the international guidelines [65].

Notably, there is knowledge lacking and different opinions regarding the prognosis of CAD patients after TAVI with current data. A retrospective study showed that revascularization in CAD patients undergoing TAVI is safe and increases one-year survival, especially in patients with multivessel CAD [66]. On the other hand, Karaduman et al. concluded that outcomes and mortality rates were similar between revascularized and non-revascularized patients after TAVI [67]. Carmona et al. established that overall and cardiac mortality were unaffected by incomplete coronary revascularization and CAD burden. However, CAD and incomplete coronary revascularization were predictors of late major/life-threatening bleeding complications in TAVI patients [68]. Compared with TAVI, the surgical approach was associated with higher 30-day complications (increased risk of bleeding, acute kidney injury, and new-onset atrial fibrillation). However, long-term outcomes were similar between the two strategies [69].

## 12. Antithrombotic Therapy Considerations after TAVI

For any post-TAVI patients, there is a huge demand for a better understanding of the optimal antithrombotic regimen to prevent valve complications. Several randomized and nonrandomized studies have examined the efficacy of various antithrombotic regimens. For instance, the POPular TAVI (Antiplatelet Therapy for Patients Undergoing Transcatheter Aortic Valve Implantation) trial investigated the role of oral anticoagulation versus oral anticoagulation plus clopidogrel on bleeding outcomes at 1 year follow up [70]. Oral anticoagulation monotherapy was associated with a lower rate of the co-primary endpoints of all bleeding and non–procedure-related bleeding than anticoagulation plus clopidogrel. At the same time, standard- or low-dose anticoagulation with either Vitamin K antagonists or oral anticoagulants after TAVR in patients without an established indication for long-term anticoagulation remains questionable. Current evidence does not support the use of short- or long-term OAC with vitamin K or NOACs after TAVR unless concomitant conditions require its use. Practice guidelines currently recommend 75 to 100 mg/d of aspirin for all patients with bioprosthetic surgical aortic valve replacement without other indications for oral anticoagulation.

## 13. Conclusions

In conclusion, the selection and management of patients undergoing aortic valve replacement for AS require careful consideration of a multitude of factors. TAVI offers a minimally invasive strategy with evidence suggesting similar, if not better, clinical outcomes when compared to SAVR. As the indications for TAVI continue to expand, there is a dire need to better understand the full spectrum of AS phenotypes. There exists a complex interplay between phenotypic variation, comorbidities, and patient risk factors that likely contributes to the variability in current post-TAVI outcomes. Furthermore, emerging evidence suggests that patient genetics may also influence aortic stenosis pathology and response to treatment. Therefore, each patient with AS should be managed with an individualized approach that takes advantage of precision medicine technologies that were previously not available. By considering each individual patient’s unique genetic makeup, comorbidities, and environmental factors, providers can better tailor treatment strategies to optimize outcomes and improve patient care in the era of advancing medical therapies and devices. Continued research and collaboration among clinicians, geneticists, and other specialists will further enhance our understanding and treatment of AS and pave the way for more personalized and effective interventions in the future.

## Figures and Tables

**Table 1:** Clinical Scenarios in which TAVI is Useful.

Clinical Scenario	Author	Y	Age, y (Mean)	Male Sex (%)	Inclusion Criteria	Results
TAVI in BAV	Halim et al. [12]	2020	74	59.1%	Patient with bicuspid or tricuspid valve undergoing TAVI	Device success was only slightly lower in the bicuspid AV group versus the tricuspid AV group (96.0% versus 96.7%; *p* = 0.004) and patients with bicuspid AV had a slightly higher incidence of intraprocedural second device implantation (1.7% versus 1.2%; *p* = 0.002). Postprocedure valve gradients were slightly higher in the bicuspid AV group (10 mm Hg versus 9 mm Hg; *p* < 0.001), with prosthetic valve area (*p* = 0.473).
TAVI in CKD	Bohbot et al. [13]	2020	78	48.1%	Severe AS (AVA < 1 cm^2^ or Indexed AVA < 0.6 cm^2^/m^2^) + one of the following: normal eGFR ≥ 60 mL/min per 1.73 m^2^; mild CKD, with an eGFR of 45 to 59 mL/min per 1.73 m^2^; moderate CKD, with an eGFR of 30 to 44 mL/min per 1.73 m^2^; and severe CKD, with an eGFR < 30 mL/min per 1.73 m^2^.	The 5-year survival rate was 71 ± 1% for patients without CKD, 62 ± 2% for those with mild CKD, 54 ± 3% for those with moderate CKD, and 34 ± 4% for those with severe CKD (*p* < 0.001). After a multivariable analysis, patients with moderate or severe CKD had a significantly higher risk of all-cause (hazard ratio [HR] [95% CI] = 1.36 [1.08–1.71]; *p* = 0.009 and HR [95% CI] = 2.16 [1.67–2.79]; *p* < 0.001, respectively) and cardiovascular mortality (HR [95% CI] = 1.39 [1.03–1.88]; *p* = 0.031 and HR [95% CI] = 1.69 [1.18–2.41]; *p* = 0.004, respectively) than patients without CKD.
TAVI in Pregnancy	Herbert et al. [14]	2019	30	0%	AS + Pregnancy in the second trimester	At 24-h postoperative follow-up, the patient reported improvement with her shortness of breath and denied complications. Four months later, she had a successful, uncomplicated spontaneous vaginal delivery of a healthy male infant.
TAVI in Cirrhosis	Peeraphatdit et al. [15]	2020	72.1	67.6%	Cirrhosis + AS undergoing either TAVR or SAVR	Society of Thoracic Surgeons (STS) score was 3.8% (1.5, 6.9,) and the median Model for End-Stage Liver Disease (MELD) score was 11.6 (9.4, 14.0). The TAVR group had similar in-hospital (1.8% versus 2.0%) and 30-day mortality (3.6% versus 4.2%) as the SAVR group. During the median follow-up of 3.8 (95% confidence interval [CI], 3.0–6.9) years, there were 63 (60%) deaths. MELD score (adjusted hazard ratio [AHR], 1.13; 95% CI, 1.05–1.21; *p* = 0.002) was an independent predictor of long-term survival.
TAVI in Nonagenarians	Deharo et al. [16]	2020	80.6	50.8%	Patients ≥ 90 with AS	TAVI was associated with lower rates of a combined outcome of all-cause death, rehospitalization for heart failure and stroke (relative risk [RR] 0.58, *p* < 0.001) in comparison with matched nonagenarians with medically treated AS.
TAVI in LVAD	Rali et al. [17]	2022	66	61.9%	Pre-existing continuous-flow LVAD undergoing TAVR or SAVR for AI	Primary composite outcome (in-hospital mortality, stroke, TIA, MI, PM implantation, vascular complications, and cardiac tamponade) occurred in 30% of patients undergoing SAVR compared with 14.3% in the TAVI group (*p* = 0.001). TAVI was associated with significantly lower odds of the composite outcome (odds ratio 0.243; 95% CI [0.06–0.97]; *p* = 0.045).

Abbreviations: AI, Aortic Insufficiency; AV, Aortic Valve; AVA, Aortic Valve Area; BAV, Bicuspid Aortic Valve; CKD, Chronic Kidney Disease; eGFR, estimated Glomerular Filtration Rate; LVAD, Left Ventricular Assistant Device; MI, Myocardial Infarction; PM, Pacemaker; SAVR, Surgical Aortic Valve Replacement, TAVI, Transcatheter Aortic Valve Implantation; TIA, Transient Ischemic Attack.

**Table 2:** Genetic Syndromes and Causes of Bicuspid Aortic Valve.

Syndrome	Genes Involved	Clinical Features
Loeys-Dietz	*TGFBR1*, *TGFBR2*, *TGFB2*, *TGFB3*, *SMAD3*	Bifid uvula, craniosynostosis, ocular disturbances
Multisystemic Smooth Muscle Dysfunction	*ACTA2*	Smooth muscle dysfunction, mydriasis
Down	21 duplication	Atrioventricular septal defects
Turner	X monosomy	Short stature, webbed neck, aortic coarctation
Velocardiofacial	22q11.2 del	Truncus arteriosus and Tetralogy of fallot
Nonhereditary bicuspid Aortic Valve	*NOTCH1*, *SMAD6*, *GATA4*, *GATA5*, *GATA6*, *ROBO4*, *MAT2A*, *ADAMTS19*	-

## Data Availability

Not applicable.
